# Cross-subject emotion EEG signal recognition based on source microstate analysis

**DOI:** 10.3389/fnins.2023.1288580

**Published:** 2023-11-28

**Authors:** Lei Zhang, Di Xiao, Xiaojing Guo, Fan Li, Wen Liang, Bangyan Zhou

**Affiliations:** ^1^Anhui Province Key Laboratory of Multimodal Cognitive Computation, School of Computer Science and Technology, Anhui University, Hefei, China; ^2^School of Electronic Information Engineering, Anhui University, Hefei, China; ^3^Key Laboratory of Civil Aviation Flight Technology and Flight Safety, Civil Aviation Flight University of China, Guanghan, China; ^4^Google Research, California, CA, United States

**Keywords:** EEG, source microstate, style transfer mapping, cross-subject, emotion recognition

## Abstract

Electroencephalogram (EEG) signals are very weak and have low spatial resolution, which has led to less satisfactory accuracy in cross-subject EEG-based emotion classification studies. Microstate analyses of EEG sources can be performed to determine the important spatiotemporal characteristics of EEG signals. Such analyses can be used to cluster rapidly changing EEG signals into multiple brain prototype topographies, fully utilizing the spatial information contained in the EEG signals and providing a neural representation for emotional dynamics. To better utilize the spatial information of brain signals, source localization analysis on the EEG signals was first conducted. Then, a microstate analysis on the source-reconstructed EEG signals is conducted to extract the microstate features of the data. We conducted source microstate analysis on the participant data from the odor-video physiological signal database (OVPD-II) dataset. The experimental results show that the source microstate feature topologies of different participants under the same emotion exhibited a high degree of correlation, which was proven by the analysis of microstate feature topographic maps and the comparison of two-dimensional feature visualization maps of the differential entropy (DE) and power spectral density (PSD). The microstate features represent more abstract emotional information and are more robust. The extracted microstate features were then used with the style transfer mapping method to transfer the feature data from the source domain to the target domain and were then used in support vector machines (SVMs) and convolutional neural networks (CNNs) for emotion recognition. The experimental results show that the cross-subject classification accuracies of the microstate features in SVMs were 84.90 ± 8.24% and 87.43 ± 7.54%, which were 7.19 and 6.95% higher than those obtained with the PSD and 0.51 and 1.79% higher than those obtained with the DE features. In CNN, the average cross-subject classification accuracies of the microstate features were 86.44 and 91.49%, which were 7.71 and 19.41% higher than those obtained with the PSD and 2.7 and 11.76% higher than those obtained with the DE features.

## Introduction

Emotional theory was proposed as early as the nineteenth century (Darwin, 1872; James, 1894), and it received enthusiastic discussion and research at that time. It also provided more inspiration and guidance for people's current research on emotional classification (Scherer, 2005). As a key factor reflecting human behavior, emotions have a direct internal connection with the way people communicate with each other. By understanding others' current emotional states, people can choose the best way to communicate to improve their communication process. The expression of emotions is a response that people make to external stimuli, and it is fixed in different areas of the brain according to the unique reactions triggered by different stimuli (Rached and Perkusich, 2013). In particular, emotional states can be mainly classified into two categories. One is the discrete model, where the emotion is thought of as discrete states such as happiness and sadness. The other is the dimensional model, which considers the emotion states to be continuous, such as the valence-arousal metric (Wu et al., 2023).

An essential goal of EEG-based emotion recognition studies is to decode EEG signals and extract more discriminating features for classifying different emotional states more effectively. Based on this goal, a vast number of studies have been conducted. Duan et al. (2013) proposed the use of differential entropy (DE) features on symmetric electrodes for the emotional recognition of subjects' electroencephalogram (EEG) signals, achieving good classification results. Padhmashree and Bhattacharyya (2022) used multivariate variational mode decomposition and multivariate modulated oscillation methods to study the emotional recognition of arousal emotions, dominant emotions, and valence emotions based on instantaneous amplitudes and instantaneous frequencies. Jie et al. (2014) used sample entropy features to distinguish positive and negative emotions in a high arousal state and subjects' emotions in different arousal states. In addition, both traditional machine learning models, such as support vector machines (Wu et al., 2023), and deep learning models, such as deep belief networks (DBNs) (Zheng and Lu, 2015) and long-short term memory (LSTM) (Wu et al., 2022), have achieved great performance for EEG-based emotion recognition. In these studies, satisfactory classification results have been achieved, indicating that EEG signals have good application prospects in the field of emotional recognition. However, the differential entropy, amplitude, and frequency features used in the above studies only contain the temporal information of EEG signals, and the spatial information of multi-channel EEG signals are not provided.

The microstate depicts the topographical map of multiple channel arrays of scalp potential, which can simultaneously result in the signals being recorded in all regions of the cerebral cortex and the topological information of the brain being fully used. In the 1980s, Lehmann et al. (1987) found that the time series of spontaneous EEG signals in the alpha frequency band maintained stability between 80 and 120 ms and that the time series of the scalp potential map suddenly changed to a new state and maintained stability again in this state. This change in the scalp potential field of the brain can reflect the momentary state of the overall activity of the underlying brain network well, and the different topographical map configurations of the brain reflect the changes in the overall and coordinated activity of the brain. These stable periods can reflect the basic steps of human brain information processing. Li et al. (2021a) used microstate analysis and geoelectrophysiological source imaging methods to study severe depression and found that microstate statistical features have good classification accuracy in identifying severe depression. Strik et al. (1997) studied the microstate of resting electroencephalograms in Alzheimer's disease and found that the duration of continuous microstates was shortened, providing a direction for the early diagnosis of Alzheimer's disease. Chen et al. (2021) used microstate analysis for emotion recognition research and, based on microstate statistical features, achieved ideal results in valence and arousal accuracy in the recognition of emotions from EEG signals. Microstate features can effectively improve the emotional recognition performance of EEG signals. Shen et al. (2020) used the microstate method of EEG to potentially characterize emotional experiences and determined that the microstate statistical features achieved better classification results in arousal and negativity in the DEAP dataset.

Currently, most studies on EEG-based emotion recognition are conducted within subjects. Due to differences in the education level, living environment, genetic factors, and the non-stationarity of EEG signals (Graimann et al., 2010), the performance of EEG-based emotion recognition tasks significantly decreases when applied across subjects. Transfer learning methods proposed by researchers in 1995 (Pan and Yang, 2010) can improve the accuracy of EEG signal-based emotion classification across subjects by reducing the differences between EEG signals from different subjects and weakening the interference of signals during data collection. Transfer learning is mainly divided into sample-based (Li et al., 2022; Zhao et al., 2023), model-based (Wang and Li, 2022; Zhao et al., 2022), and feature-based (Li et al., 2021b; Khalil et al., 2022) methods. Feature-based transfer learning methods minimize the distance between the source and target domains (Dai et al., 2007), enabling the model trained on the source domain data to perform well in the target domain, thereby improving the accuracy of cross-subject emotion classification.

During the experiment, source localization analysis was first conducted on the OVPD-II preprocessed data, and source microstate analysis was then performed on the traced data after the global field power was calculated. Four statistical features of the source microstate prototype coverage, duration, occurrence rate, and microstate sequence transition probability were calculated for the participants. In this study, source microstate features were applied to cross-subject EEG-based emotion recognition research for the first time. The calculated source microstate statistical features were put into the style transfer mapping method for feature transfer, and the transferred feature data were put into a support vector machine (SVM) and convolutional neural network (CNN) for cross-subject emotion classification.

## Materials and methods

### Introduction to the dataset

As an important human sense, olfaction constantly affects people's daily lives. OVPD-II is a physiological signal database based on olfactory odors and video induction, and it was developed from the OVPD dataset (Xue et al., 2022) and other datasets from Anhui University. It includes two sub-datasets: video stimulation data and video with odor stimulation data. Thirty representative Chinese film clips were selected as video stimuli from 70 films for the experiment. At the same time, 10 common odors that can arouse the emotions of the participants and are consistent with the video were chosen as olfactory stimuli. These odors include four that can evoke positive emotions (rose, orange, lavender, and toilet water), four that can generate negative emotions (a cleaning agent, alcohol, vinegar, and ink), and two neutral odors that have little effect on emotions (odorless pure water and air).

**Figure 1:**
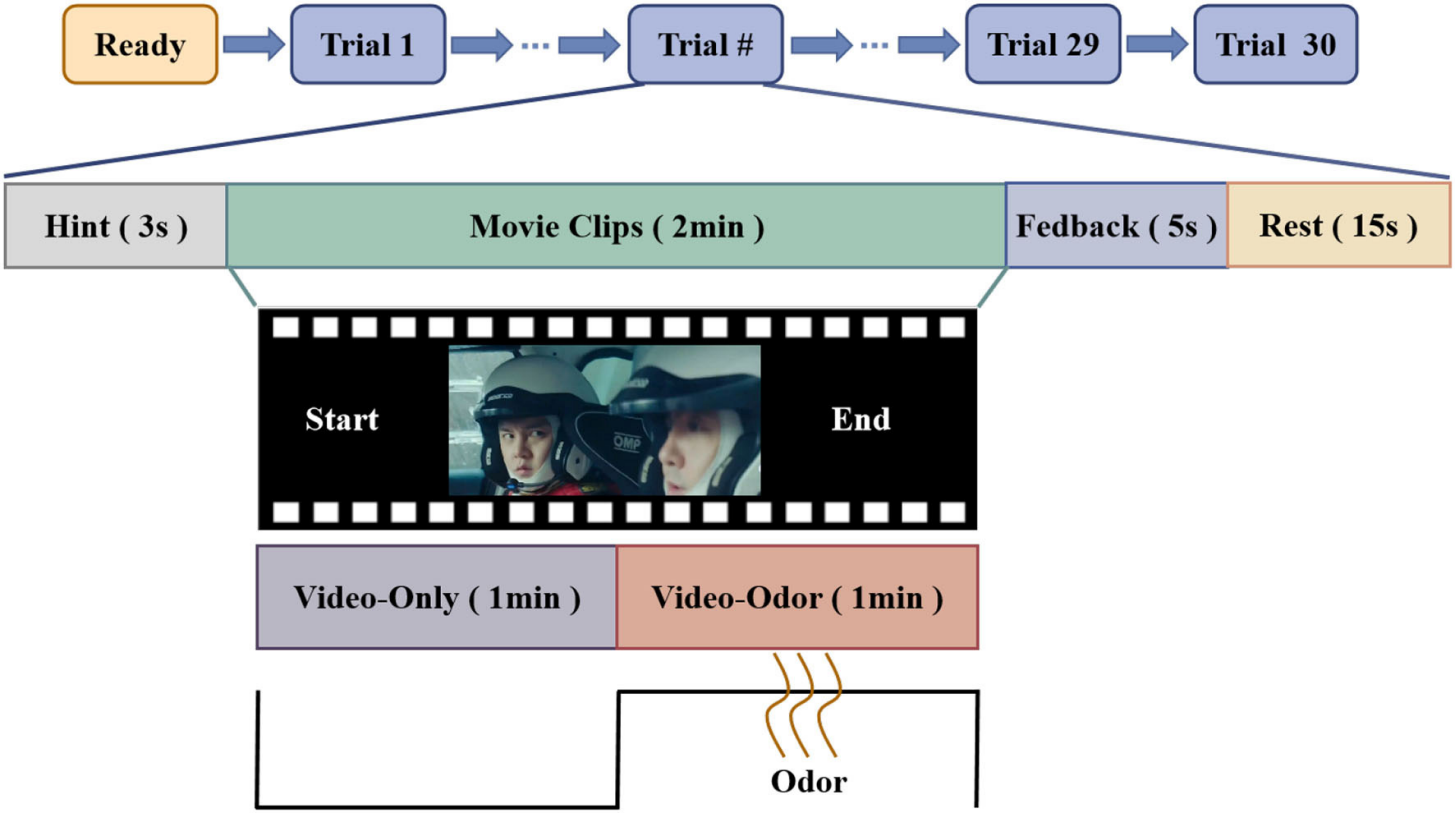
OVPD-II dataset EEG signal acquisition paradigm. The paradigm mainly consists of 4 modules: the hint module that was implemented for 3 s, the emotion evoking module that was implemented for 2 min, the feedback module that was implemented for 5 s, and the rest module that was implemented for 5s. In particular, the emotion evoking module consists of two patterns, i.e., in the first minute of the video stimuli, we only use video stimuli to evoke the emotions of the subjects, while in the last minute of the video stimuli, we applied both a video stimuli and odor to evoke the emotions of the subjects. In addition, in the feedback module, all subjects were asked to report their feedback from −7 to 7.

As there are individual differences in the emotional tendencies toward the same odor, each participant underwent two sets of repeated odor tests before the formal experiment started. Finally, the odors that can induce the same emotion as that obtained from the video stimulus are selected based on the emotions induced by different odors on the participants and the emotions aroused by the video. Each experiment starts with a 3-s prompt, and the participants need to watch a 2-min video clip. After watching the video for 1 min, the experimenter placed an odor related to the content of the clip approximately 2 cm below the participant's nostrils for 1 min. To minimize the influence of the previous odor on the emotions, there was a transition time of 1.5 s between two adjacent odor stimuli. After watching the video, there was a feedback time of 5 s and a rest time of 15 s. The experimental paradigm is shown in Figure 1.

**Figure 2:**
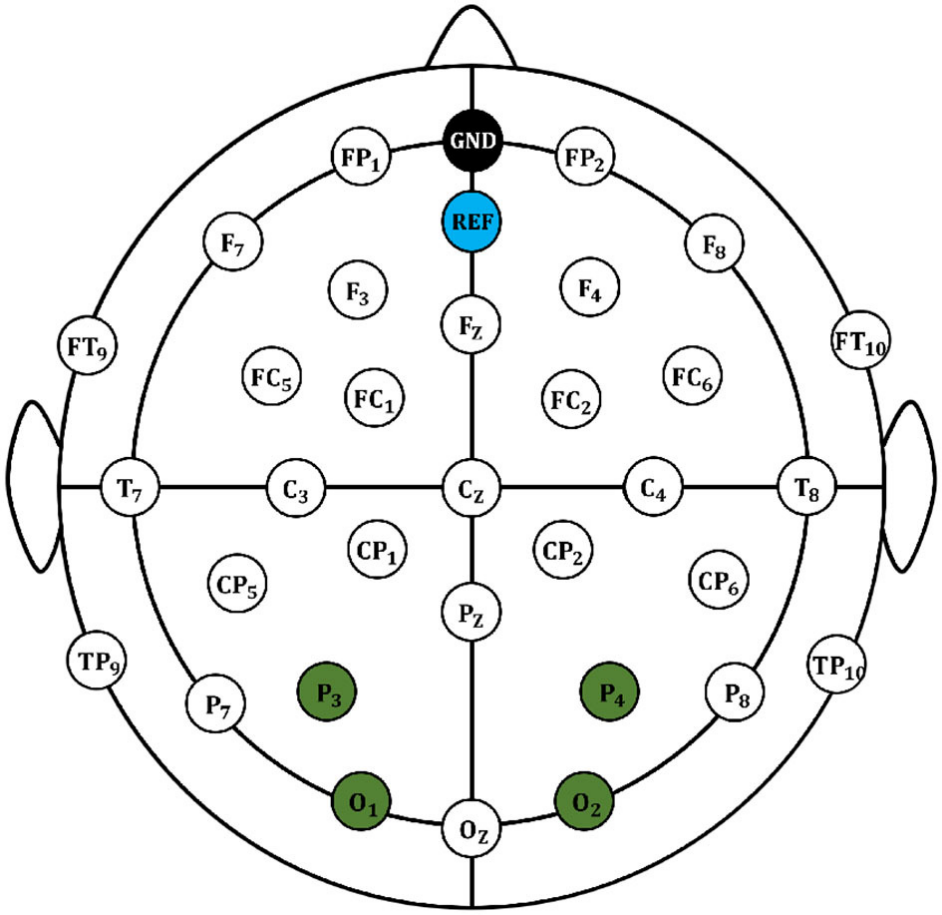
Electrode distribution map of EEG signal acquisition.

Thirteen mentally and physically healthy students aged between 18 and 27, without visual, auditory, or olfactory impairments, participated in the experiment. These students included 7 males and 6 females. Moreover, a 32-channel electrode distribution, in which four electrode channels (O1, O2, the P3, and P4) were used to record the participants' eye signals, were utilized in the experiment, and the remaining 28 channels were used to record the participants' EEG signals, as shown in Figure 2. The EEG data were collected at a frequency of 250 Hz, and after watching the video, the participants needed to evaluate their emotions based on their own feelings. The evaluation criteria were marked from 3 to 7 for positive emotions, from −2 to 2 for neutral emotions, and from −3 to −7 for negative emotions. Then, the obtained two-dimensional emotional labels were mapped to a discrete emotional model of positive, neutral, and negative emotions.

### Data preprocessing

**Figure 3:**
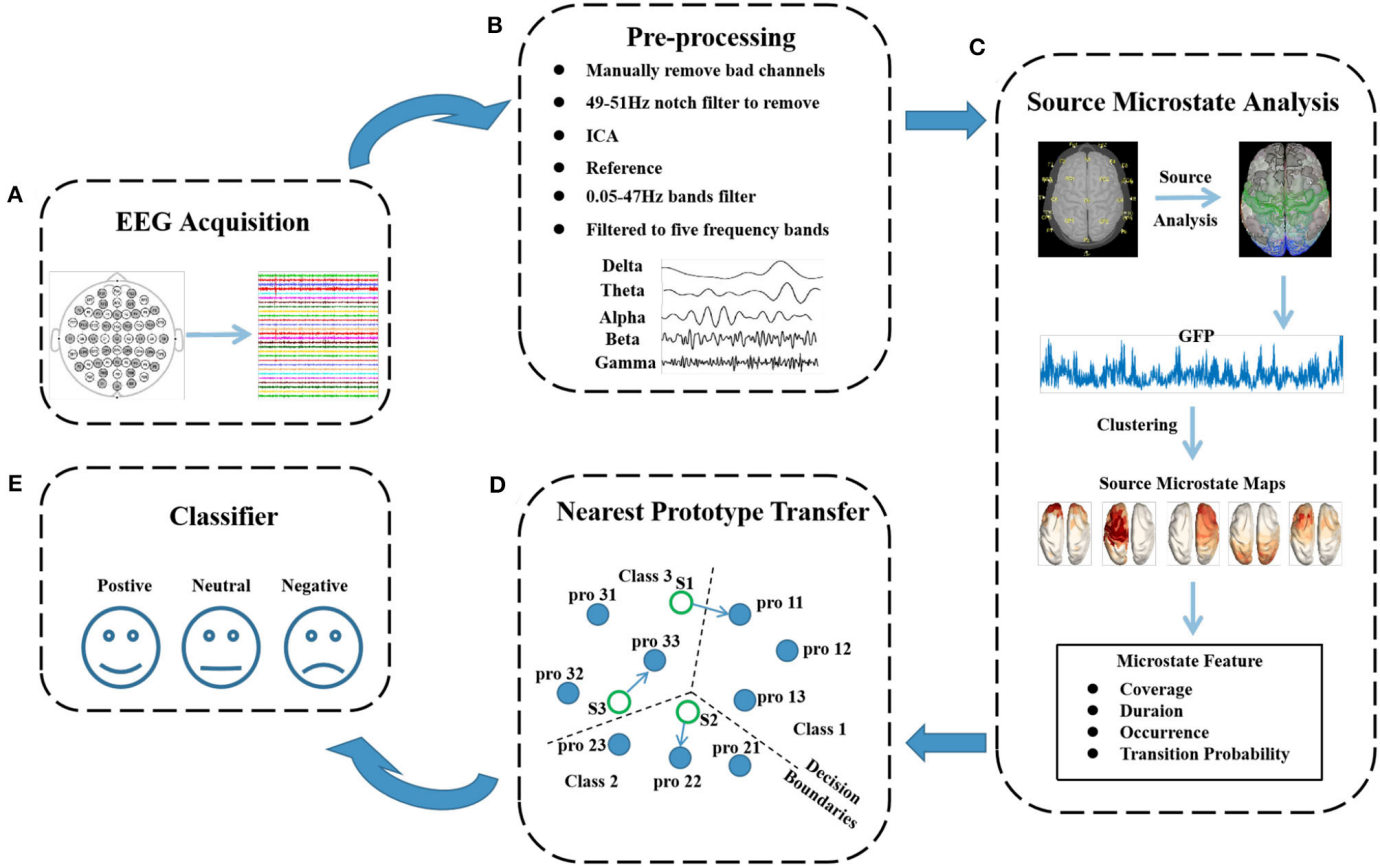
Flowchart of cross-subject emotion recognition using the microstate features. The flowchart consists of 5 modules: **(A)** recording of the emotional EEG signals; **(B)** preprocessing of the EEG signals, where interpolation for bad channels, notch filtering, artifact removal, reference, and bandpass filtering were successively performed; **(C)** extraction of the microstate features of the EEG signals, such as coverage and duration; **(D)** feature transfer process based on the style transfer mapping method; and **(E)** emotion classification based on SVM or CNN methods.

During the experiment, the OVPD-II dataset was recorded shown in Figure 3A, and preprocessed before the microstate features and EEG source localization were extracted. First, the four electrode channels used for collecting eye signals were removed, and only the data from the 28 channels used for collecting EEG signals were used. Then, bad electrodes were manually interpolated. The EEGLAB toolbox in MATLAB was used for preprocessing (Delorme and Makeig, 2004). First, a notch filter with a frequency of 49–51 Hz was used to remove power line interference. Then, a bandpass filter was used to retain the EEG data in the frequency range of 0.05–47 Hz. The data after filtering were re-referenced, and independent component analysis was used to remove eye movement artifacts from the EEG signals. Finally, the denoised EEG data were divided into five frequency bands: delta, theta, alpha, beta, and gamma, as shown in Figure 3B.

### Source microstate analysis

As shown in Figure 3C, the source microstate analysis was employed to extract the microstate information. The EEG source analysis method measures the potential at different locations on the brain to simulate the electric currents generated by the neuronal activity in the brain. It calculates the optimal current source in the brain that best fits these EEG data, and the activity during the fitting period can be approximated well by a current dipole (Helmholtz, 1853). The cortical surface of the brain is divided into a very detailed grid as the potential location of sources in EEG source analysis methods. The brain source signal is calculated by considering the direction and magnitude of the source of all grid points perpendicular to the cortical surface (Michel et al., 2004). In this experiment, a distributed source localization method was used (Jun et al., 2019). It is assumed that there are *m* sensors and *n* samples in the EEG signal. Moreover, *AϵR*^*m*×*n*^ represents the measured scalp signal, *d* represents the number of dipoles in the model, *BϵR*^*m*×*d*^ represents the lead field matrix in the model, θ*ϵR*^*d*×*n*^ represents the dynamic sources of *d* dipoles and *n* EEG samples, and ω_1_ represents the random fluctuations in the sensor space. The specific algorithm for the inverse problem of distributed source localization is as follows:


(1) $$\begin{array}{l} A=B\theta +\omega_{1} \end{array}$$

Standardized low-resolution brain electromagnetic tomography (sLORETA) uses the collected EEG data to achieve EEG source localization analysis and create a standardized low-resolution brain source model for further study of the brain's processing of signal stimulation (Scharmüller et al., 2012). Assuming *X* represents the Laplacian operator in discrete space, *BϵR*^*m*×*d*^ represents the lead field matrix in the model, *W* represents the weighting matrix, and *d* represents the number of dipoles in the model, the specific calculation method of *W* is:


(2) $$\begin{array}{l} W=X\times diag\left(||B_{1}||,\;||B_{2}||,\;\ldots ||B_{d}||\right) \end{array}$$

The sLORETA method further considers the postprocessing of the standardized current density power. Assuming θ*ϵR*^*d*×*n*^ represents the dynamic source of *d* dipoles and *n* EEG samples, *D*_θ_ represents the variance of the estimated current density; then, the specific algorithm for θ*ϵR*^*d*×*n*^ and *D*_θ_ is:


(3) $$\begin{array}{l} \theta_{l}^{{\;}^{\prime }}=\;\theta_{l}^{T}{\left\{{D_{\theta }}_{ll}\right\}}^{-1}\theta_{l} \end{array}$$

(4) $$\begin{array}{l} D_{\theta }=W^{-1}A^{T}{\left(AW^{-1}A^{T}\right)}^{+}A \end{array}$$

In microstate analysis, the electric field topography results at the local maximum of the global field power (GFP) (Koenig et al., 2002) curve are considered the discrete states of the EEG signals (Brunet et al., 2011). Assuming *C* is the number of electrodes in the EEG signal, 28 channels are used in the OVPD-II dataset to record the EEG signals of participants. *V*(*i, t*) represents the instantaneous potential of the *i*−*th* electrode at time *t*, and *V*(*t*) represents the mean value of the potential of all electrodes at time *t*. The calculation method of global field power is as follows:


(5) $$GFP=\;\sqrt{\frac{\sum_{i}^{k}{\left(V_{i}(t)-\overset{-}{V}(t)\right)}^{2})}{C}}$$

The K-means clustering algorithm (Arthur and Vassilvitskii, 2007) uses the peak values of the GFP of the EEG signals as the original topography maps for clustering. Then, several scalp topography maps are randomly selected from all original topography maps as template maps for microstate classes. The spatial correlation between each template topography map and the original topography map of the EEG data is calculated. Based on the calculated spatial correlation sequence, the global explained variance (GEV) of each microstate class topography map is calculated to measure the explained variance of the selected microstate topography maps for the entire EEG data (Khanna et al., 2015). Assuming μ_1_, μ_2_ to μ_*n*_ represent the mapping of *n* clustering templates, where μ_*j*_ represents the *j*−*th* clustering template, *x*^(*i*)^ is the original topographic map of EEG data, and *l*^(*i*)^ is the label of the nearest microstate template to the original topographic map. The calculation method is as follows:


(6) $$\begin{array}{l} I^{\left(i\right)}\;:=\;{argmin}_{j}||X^{i}-\mu_{j}|{|}^{2} \end{array}$$

(7) $$\begin{array}{l} \mu_{j}\;:=\;\frac{\overset{n}{\underset{i\;=1}{\sum }}\left\{l^{i}\;=j\right\}X^{i}}{\overset{n}{\underset{i\;=1}{\sum }}\left\{l^{\left(i\right)=j}\right\}} \end{array}$$

For the selection of the optimal number of microstate clusters, the GEV and cross-validation (CV) criteria (Pascual-Marqui et al., 1995) are used in this study to select an appropriate number of microstate clusters. The GEV is used to calculate the correlation between the EEG signals and the microstate prototypes at a certain time, quantifying the degree of match between the EEG data and the assigned microstates. The higher the GEV value is, the better the fit between the microstates and the data. The CV criterion is used to measure the residual noise between the EEG signals and the microstate topography maps. The smaller the value is, the more the clustering of the microstate numbers can explain the EEG signals (Murray et al., 2008). Assuming *x*_*i*_ is the EEG signal at time *i*, *p*_*li*_ represents the label of the microstate topography map of the *i*−*th* EEG sample, *GFP*_*i*_ represents the standard deviation of all electrodes in the *i*−*th* EEG sample, where *C* represents the number of EEG channels, *K* represents the number of microstates, and ${\hat{\sigma }}^{2}$ represents the variance of the residual noise. The specific calculation methods of the GEV and CV criterion are as follows:


(8) $$\begin{array}{l} {GEV}_{i}=\;\frac{{\left(Corr\left(X_{i\;},\;p_{li}\right)\;•\;{GFP}_{i}\right)}^{2}}{\overset{N}{\underset{i^{{\;}^{\prime }}}{\sum }}{GFP}_{i^{{\;}^{\prime }}}^{2}} \end{array}$$

(9) $$\begin{array}{l} CV=\;{\hat{\sigma }}^{2}•{\left(\frac{C-1}{C-K-1}\right)}^{2}\;,{\hat{\sigma }}^{2}=\;\frac{\overset{N}{\underset{n}{\sum }}X_{i}^{T}X^{i}-{\left(a_{li}^{T}X_{i}\right)}^{2}}{N\left(C-1\right)} \end{array}$$

After an appropriate source microstate prototype is selected, the source microstate topographic map is inputed back to the original data, and the statistical features of the source microstate, including the time coverage, duration, occurrence, and transition probabilities between the source microstates, are calculated. The time coverage of the source microstate template refers to the proportion of the source microstate topographic map active in the entire analyzed EEG data. The duration refers to the average length of each source microstate topographic map in milliseconds of EEG data sequences. The occurrence of the source microstate represents the average frequency of occurrence of a source microstate class per second. It is calculated by dividing the number of segments belonging to a source microstate class by the total duration of the analysis data in seconds. The transition probabilities refer to the transition probability between any two given source microstate templates.

### Style transfer mapping

As shown in Figure 3D, the style transfer mapping is introduced to map the target domain EEG features into source domain to narrow the difference of data distribution between different domains. In transfer learning, we use the training set data as the source domain and the test set data as the target domain. The style transfer mapping method maps the data from the source domain to the target domain through affine mapping, reducing the distance between the source and target domains (Zhang and Liu, 2012). This causes the classification model to be more familiar with the target domain data, leading to better classification results. It is assumed that the target domain data are represented as $X=\left\{x_{i}\in R^{M}|i=1,2,3,\ldots N\right\}$ and that the source domain data are represented as $Y=\left\{y_{i}\in R^{D}|i=1,2,3,\ldots N\right\}$, where *M* represents the feature dimension and *N* represents the number of data points in the target domain. Assuming *x*_*i*_ is mapped to *y*_*i*_ under the condition that the confidence level is set to *f*_*i*_∈[0, 1], concept drift may occur after mapping. The style transfer mapping transforms *y*_*i*_ back to *x*_*i*_ through the inverse function *Ay*_*i*_+*b* and learns the transfer matrix *x*_*i*_ by minimizing the weighted square error. The specific calculation method is as follows:


(10) $$\overset{min}{A\in R^{M\times M},b\in R^{M}}\sum_{i=1}^{N}f_{i}||Ay_{i}-x_{i}|{|}_{2}^{2}+\beta ||A-I|{|}_{F}^{2}$$

β is used to balance the degree of data transfer and non-transfer to avoid excessive transfer. A value of β that is too small may result in excessive transfer, while a larger value may not be conducive to transfer. The calculation method of β is:


(11) $$\begin{array}{l} \beta =\;\overset{\sim }{\beta }Tra\left(f_{i}y_{i}y_{i}^{T}\right) \end{array}$$

After cross-validation, the value of $\overset{\sim }{\beta }$ can be chosen from the range of [1,3]. During the style transfer mapping, the nearest prototype model (Bezdek and Kuncheva, 2001) is used for destination mapping. The source domain data are clustered using the K-means clustering algorithm, and the cluster centers serve as the prototypes required for the experiment. The specific calculation method is as follows:


(12) $$\begin{array}{l} p_{ij}=\;\epsilon R^{M},j=i,\ldots ,n_{i},i=1,\ldots ,M \end{array}$$

### Experimental settings

**Figure 4:**
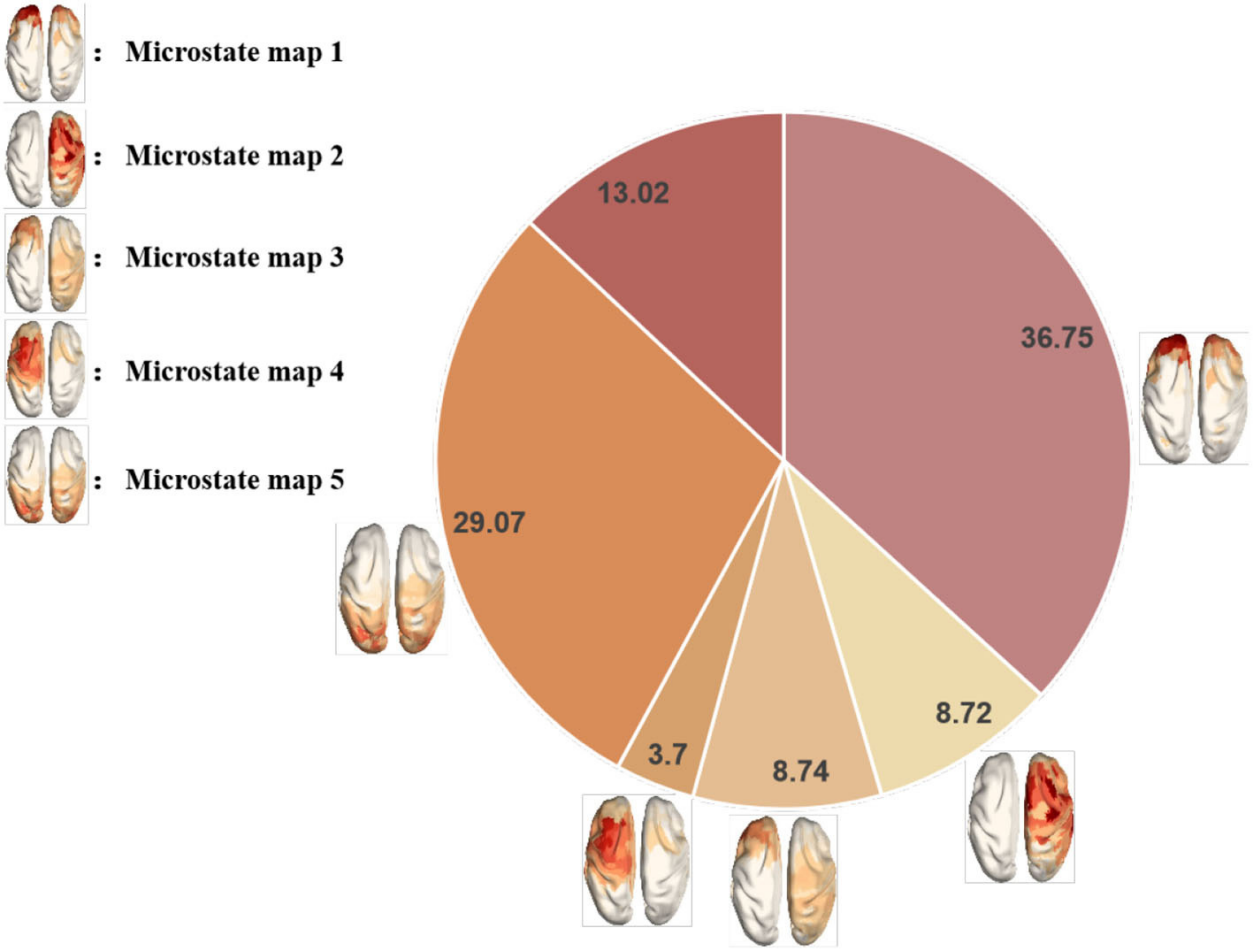
Topological map of the source microstate in the pure video stimulus dataset.

In this experiment, the source microstates of the preprocessed OVPD-II dataset were first analyzed. We first calculated the GFP of the five frequency bands of the EEG data for 13 subjects in the dataset. Then, we used the K-means clustering method to cluster the GFP for source microstate clustering and selected an appropriate number of source microstate clusters based on the GEV and CV criteria. Through calculation, we finally selected 5 source microstate topologies that can explain approximately 85% of the EEG data information in the OVPD-II dataset, as shown in Figure 4, which is the source microstate topology of the OVPD-II dataset in the alpha band. We fitted the EEG data based on the selected source microstate prototypes and finally smoothed the fitted data over time. Then, we calculated the statistical features of the 5 source microstates, including the time coverage, duration, occurrence, and transition probabilities. The dimensions of the coverage range, duration, and occurrence frequency are consistent with the number of microstate categories, all of which are row vectors of size 1 × 5. The dimension of the transition probability is a data matrix of size 5 × 5. Therefore, the feature dimension of a single frequency band sample is 5 × (5+3) = 40 dimensions. The EEG data features of the 5 frequency bands are fused, and the feature dimension after frequency band fusion is 200 dimensions.

The OVPD-II dataset contains EEG data from 13 participants. We employed a multi-source domain style transfer mapping method (Li et al., 2019), using 12 subjects in the dataset as the source domain and the remaining subject as the target domain. The 12 subjects were trained using SVM and CNN (shown in Figure 3E), and the trained model was tested using verification data. The best performing model was selected, and the source domain data used to train the model were identified. The target domain data and source domain data were migrated using the style transfer mapping method, and the migrated data were classified using a classifier. The experimental confidence level *f*_*i*_ value was determined to be 0.8, and β was determined to be 0.2 through cross-validation analysis.

For the parameter setting of a classification model, choosing the linear kernel function for the SVM classifier can achieve the highest classification accuracy. Moreover, the grid search method from the parameter pool, *P*_*v*_*alue* = {0.01*k*, 0.1*k, k* |*k* = 1, 2, ..., 9}, was adopted to choose the optimal regulation parameter *C* and the kernel function hyperparameter gamma. For the CNN experiment, a single-layer CNN was used, with the hyperparameters listed in Table 1. The experimental environment was built on a Windows 10 PC with Core (TM) i7-8700 CPU and 16 GB memory, and the computing environment was MATLAB 2019b.

**Table 1:** Setting of the hyperparameters in the convolutional neural networks.

**Hyper parameter**	**Values**
Learning rate	0.005
Batch size	16
Learning decay coefficient	0.8
Regularization	0.001
Epoch	100

## Results and discussion

### Analysis of the source microstate features

**Figure 5:**
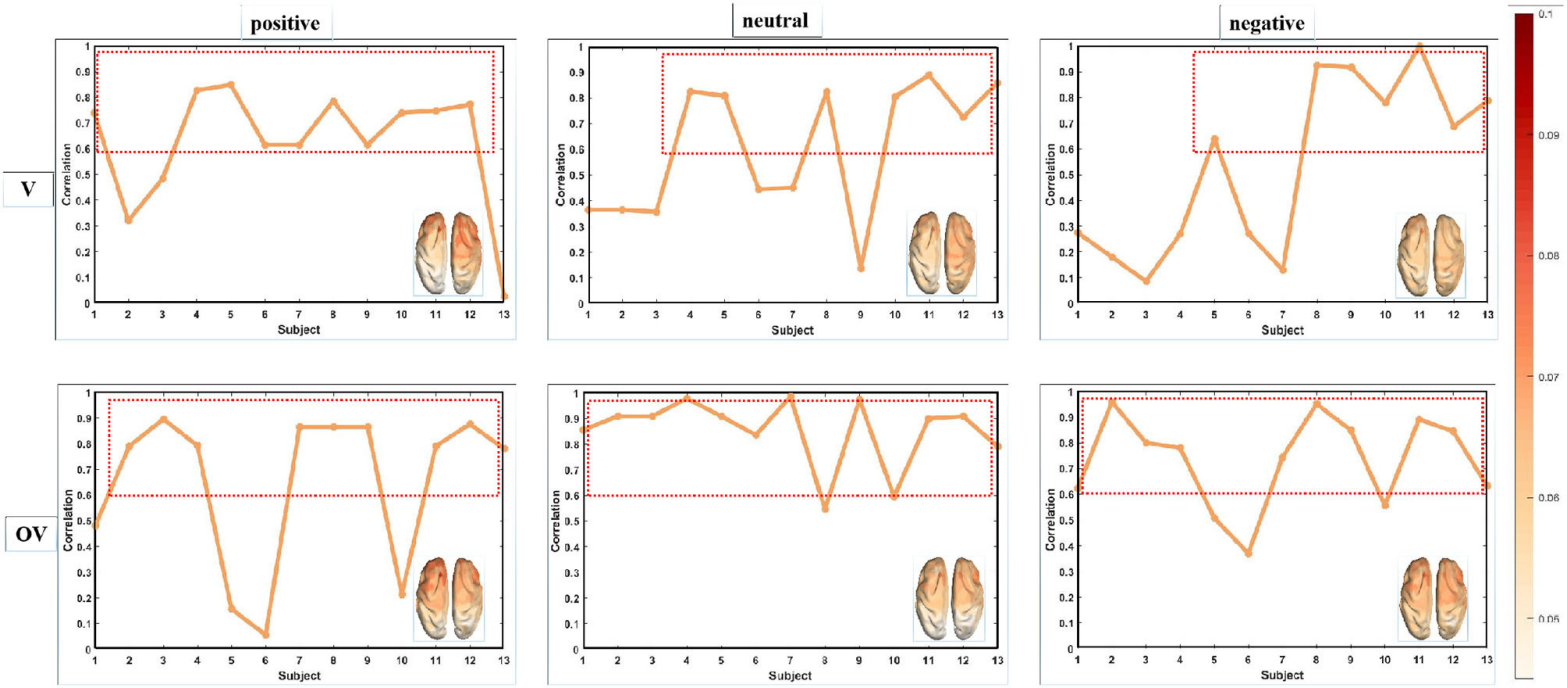
Topographic map of the microstate features in the OVPD-II video stimulus dataset.

In the experiment, non-overlapping 1-s sliding windows were used to extract the source microstate features from the subjects in the OVPD-II dataset, and the topological maps of the source microstates were analyzed. By calculating the GFP values of each subject under each emotion, the source microstate prototypes that could explain approximately 70% of the EEG information were selected as representative topological maps for each subject. We calculated the common active areas of the representative microstate topological maps of 13 subjects under three emotions and drew a typical source microstate prototype that could represent the dataset. The Pearson correlation coefficients between the 13 subjects and the source microstate prototype were calculated, as shown in Figure 5, where “V” represents the video stimuli and “OV” represents odor with video stimuli. The results of the experiment show that the topological maps of the source microstates extracted from different subjects under the same emotion had high correlation. We believe that this is because the microstates reflect the overall coordinated changes in brain activity. Regarding the clustering of the source microstates of all 13 subjects in the dataset, the selected 5 topological maps could explain 85% of the EEG data, and the differences in the active areas of different subjects were ignored in the selected microstate templates under the same emotion. Therefore, the statistical features of the source microstates from different subjects have high robustness.

Under video stimulation, the active regions of the positive emotion source microstate topography were mainly concentrated in the frontal and parietal lobes, those of the neutral emotion were concentrated in the frontal, parietal, and occipital lobes, and those of the negative emotion were concentrated in the frontal and parietal lobes. After adding odor stimulation that could induce the same emotion in the subjects, the activity level of the source microstate in the frontal lobe increased for positive and negative emotions, while the odor stimulation for the neutral emotion was colorless and tasteless (pure water and air), and the active region of the source microstate topography did not show a significant enhancement. This indicates that adding odor stimulation can induce stronger emotional responses in the subjects, and this result is consistent with the experimental conclusion (Xue et al., 2022).

### Non-transfer cross-subject emotion recognition

Moreover, the DE and power spectral density (PSD) features of non-overlapping 1-s sliding windows are calculated in this experiment, where the DE features are calculated as:


(13) $$\begin{array}{l} h(x)\text{​}=-\text{​}\int_{-\infty }^{\infty }\frac{1}{\sqrt{2\pi \sigma^{2}}}e^{-\frac{{\left(x-\mu \right)}^{2}}{2\sigma^{2}}}\log(\frac{1}{\sqrt{2\pi \sigma^{2}}}e^{-\frac{{\left(x-\mu \right)}^{2}}{2\sigma^{2}}})\text{d}x\\ \;=\frac{1}{2}\log(2\pi e\sigma^{2}) \end{array}$$

The time series *X* follows a Gaussian distribution *N*(μ, σ^2^).

**Figure 6:**
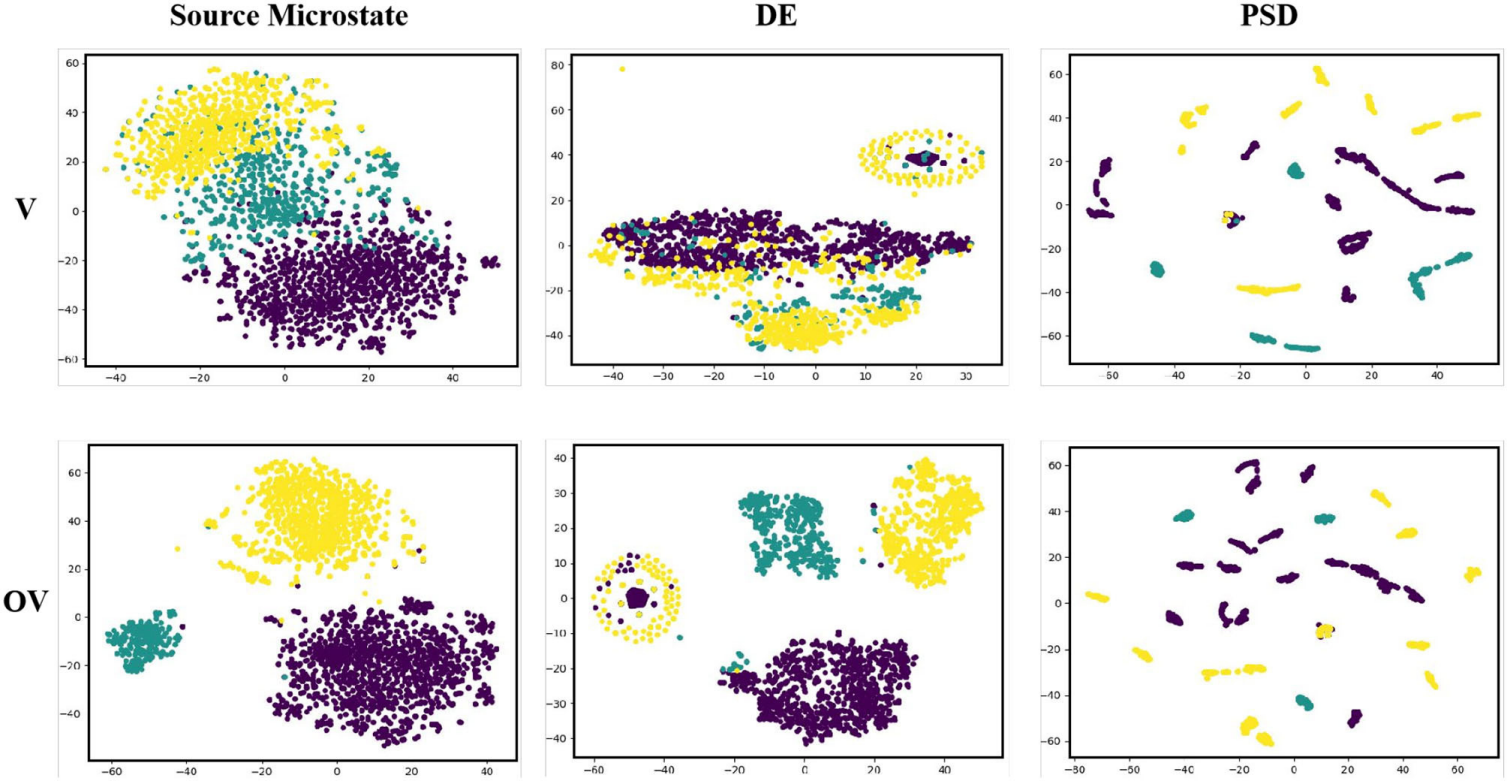
2D visualization of the microstate, differential entropy, and power spectral density features.

The t-distributed stochastic neighbor embedding (t-SNE) function was used to reduce the dimensionality of the data features and draw a two-dimensional visualization feature map. Figure 6 shows the feature visualization map of Subject HH, where the purple dots represent positive emotions, the yellow dots represent neutral emotions, and the green dots represent negative emotions. The results of the experiment show that the two-dimensional visualization feature map of the source microstate was different from those of DE and PSD. The clustering distribution boundaries of the features obtained were more obvious. In addition, it was observed that there was more obvious clustering of the positive and neutral emotion samples in DE and clustering of the positive, neutral, and negative emotion samples in PSD, which may be due to the difference in the induced materials. This phenomenon did not occur in the source microstate features we extracted, and we believe that this feature can capture more abstract emotional information compared to DE and PSD.

Before performing data transfer, we separately input the extracted three types of features into SVM and CNN for emotion classification. Table 2 shows the cross-subject classification results of the source microstate features, DE and PSD before style transfer mapping under pure video stimulation and video-odor stimulation. In SVM, the average classification accuracy of the source microstate was the best, with 60.95 and 64.79%, respectively, which were 4.06 and 6.14% higher than the accuracy of DE and 3.84 and 5.83% higher than that of PSD. In CNN, the average classification accuracy of the source microstate was 65.09 and 66.39%, respectively, which was 9.59 and 11.73% higher than that of DE and 2.99 and 4.08% higher than that of PSD. The experimental results show that cross-subject emotion recognition based on source microstate features achieved good classification results in both SVM and CNN.

**Table 2:** Cross-subject classification accuracy without transfer learning (%).

**Feature**	**V**		**OV**	
	**SVM**	**CNN**	**SVM**	**CNN**
Source microstate	**60.95**	**65.09**	**64.79**	**66.39**
DE	56.89	55.50	58.65	54.66
PSD	57.11	62.10	58.96	62.31

The bold values mean the highest of each column.

### Cross-subject emotion recognition based on style transfer mapping

We used the extracted source microstate features, DE and PSD to perform nearest prototype feature transfer mapping. The transferred features were separately input into SVM and CNN for cross-subject emotion classification under video stimuli. The recognition accuracy of the three features is shown in Table 3. The experimental results show that the source microstate features had the highest recognition accuracies in cross-subject classification in both SVM and CNN, with average recognition accuracies of 84.90 and 86.44% for 13 subjects, which were 1.28 and 2.7% higher than those obtained with the DE, and 7.19 and 7.13% higher than those obtained with the PSD.

**Table 3:** Cross-subject recognition accuracy based on source microstate features, differential entropy, and power spectral density under video stimuli (%).

**Participant**	**SVM**			**CNN**		
	**Source microstate**	**DE**	**PSD**	**Source microstate**	**DE**	**PSD**
CL	93.44	88.76	85.07	98.83	92.96	94.37
CSY	84.22	81.65	60.65	88.68	83.31	72.50
HH	82.15	83.78	78.96	77.18	86.57	83.80
LHY	87.25	82.69	81.34	53.85	88.69	83.80
LWX	89.90	80.05	88.81	99.39	88.45	86.62
RJ	89.65	93.08	81.34	63.94	81.925	83.10
SX	88.33	83.48	91.79	98.26	82.96	92.25
WCQ	92.36	84.86	81.25	92.47	84.32	87.04
WJQ	62.53	73.49	60.30	97.18	84.37	88.73
XD	85.08	86.47	84.78	91.94	59.58	38.06
XJ	83.43	76.71	63.48	87.59	85.185	63.889
XMX	89.82	85.86	86.49	97.922	83.59	62.47
YXK	75.60	86.23	65.94	76.48	86.71	94.37
Mean	**84.90**	83.62	77.71	**86.44**	83.74	79.31
Std	8.24	5.04	11.10	14.53	7.85	16.19

The bold values mean the highest of each column.

**Figure 7:**
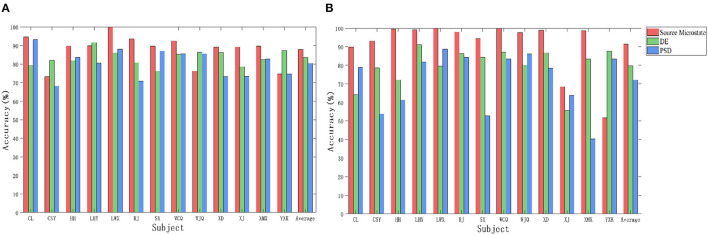
The cross-subject recognition accuracy of the three features under odor plus video stimuli. **(A)** Support Vector Machine. **(B)** Convolutional Neural Network.

Figure 7 shows the cross-subject recognition accuracy of the three features under odor with video stimuli. In SVM and CNN, the recognition accuracies of the source microstate were 87.43 and 91.49%, respectively, which were 1.79 and 18.77% higher than those obtained with the DE and 6.95 and 19.82% higher than those obtained with the PSD. The experimental results show that the source microstate achieved ideal classification results in both cross-subject emotion recognition under video and odor with video stimuli. Moreover, the cross-subject recognition accuracy based on source microstate features under odor plus video stimuli was 2.53 and 5.05% higher than that under video stimuli in SVM and CNN, respectively, indicating that the addition of odor stimuli induced stronger emotions in the subjects.

The experimental results show that the combination of the microstate features and style transfer mapping achieved promising results in cross-subject emotion classification. After feature transfer, the cross-subject emotion classification accuracy significantly improved compared to that before transfer. Specifically, the EEG data with video stimuli increased by 23.95 and 21.35% in SVM and CNN, respectively. The cross-subject emotion classification accuracy with video and odor stimuli increased by 22.64 and 25.10% in the two classifiers, respectively. This indicates that the combination of style transfer mapping and source microstate features can be effectively applied to cross-subject emotion recognition research based on EEG signals.

## Conclusions and future works

Based on the problem of low recognition accuracy in cross-subject emotion recognition using EEG signals, the robust source microstate and style transfer mapping methods are combined in this study, and they are applied to the odor-video-induced physiological signal database. The source microstate reflects the overall coordinated changes in brain activity. When conducting source microstate analysis based on the dataset, we ignored the differences in the active areas of different subjects under the same emotion, resulting in a high correlation between the source microstate topological maps of different subjects and certain robustness in the statistical features of source microstates from different subjects. Furthermore, the two-dimensional visualization feature map of the source microstate, in contrast to the feature maps of DE and PSD, has more obvious distribution boundaries and can characterize more abstract emotional information than DE and PSD, showing significant advantages in cross-subject emotion recognition research.

After combining the source microstate feature and style transfer mapping method, the cross-subject emotion recognition accuracy was significantly improved. The recognition accuracies of the source microstate after style transfer in pure video were 84.90 and 86.44% in SVM and CNN, respectively, which were 23.95 and 21.35% higher than the cross-subject recognition accuracies before transfer. The recognition accuracies of the source microstate after style transfer in the odor plus video were 87.43 and 91.49% in SVM and CNN, respectively, which were 22.64 and 25.10% higher than the cross-subject recognition accuracies before transfer. Adding odor stimulation increased the emotion recognition accuracies of the subjects by 2.53 and 5.05% compared to video stimulation, indicating that stronger emotions were induced in the subjects with the addition of odor stimulation.

In the current work, we focus on the emotion recognition of EEG single modalities, while other modalities, such as facial expressions, speech signals, and eye-tracking signals, also contain rich emotional information. OVPD-II is a physiological signal database for emotion recognition based on odor video, in which 4 channels record eye-tracking signals during emotional induction. In future work, we will attempt to fuse eye-tracking signal features and source microstate features for multi-modal cross-subject emotion recognition research. However, brain connectivity is a common and effective analysis method for EEG signals, and we will study the correlation and difference between the brain connectivity functions and the EEG microstates for emotion recognition tasks. We will also test the effectiveness of the EEG microstate features in other public datasets by combining deep learning methods.

## Data availability statement

The original contributions presented in the study are included in the article/supplementary material, further inquiries can be directed to the corresponding author.

## Ethics statement

The studies involving humans were approved by Biomedical Ethics Committee, Anhui University. The studies were conducted in accordance with the local legislation and institutional requirements. The participants provided their written informed consent to participate in this study.

## Author contributions

LZ: Methodology, Software, Writing—review and editing. DX: Data curation, Formal analysis, Software, Writing—original draft. XG: Data curation, Methodology, Supervision, Writing—review and editing. FL: Methodology, Supervision, Writing—review and editing. WL: Investigation, Supervision, Writing—review and editing. BZ: Methodology, Software, Supervision, Validation, Writing—review and editing.
